# Quinoline Photobasicity: Investigation within Water‐Soluble Light‐Responsive Copolymers

**DOI:** 10.1002/chem.202003815

**Published:** 2020-12-04

**Authors:** Maria Sittig, Jessica C. Tom, Johanna K. Elter, Felix H. Schacher, Benjamin Dietzek

**Affiliations:** ^1^ Department of Functional Interfaces Leibniz Institute of Photonic Technology Jena Albert-Einstein-Strasse 9 07745 Jena Germany; ^2^ Institute of Physical Chemistry and Abbe Center of Photonics Friedrich-Schiller-University Jena Helmholtzweg 4 07743 Jena Germany; ^3^ Institute of Organic Chemistry and Macromolecular Chemistry (IOMC) Friedrich Schiller University Jena Humboldtstrasse 10 07743 Jena Germany; ^4^ Jena Center for Soft Matter (JCSM) Friedrich Schiller University Jena Philosophenweg 7 07743 Jena Germany

**Keywords:** light-responsive polymers, photoswitch photobasicity, quinoline, transient absorption spectroscopy

## Abstract

Quinoline photobases exhibit a distinctly higher p*K*_a_ in their electronically excited state than in the ground state, thereby enabling light‐controlled proton transfer reactions, for example, in molecular catalysis. The absorption of UV light translates to a p*K*_a_ jump of approximately 10 units, as established for small‐molecule photobases. This contribution presents the first synthesis of quinoline‐based polymeric photobases prepared by reversible addition‐fragmentation chain‐transfer (RAFT) polymerization. The integration of quinolines as photobase chromophores within copolymers offers new possibilities for light‐triggered proton transfer in nanostructured materials, that is, in nanoparticles, at surfaces, membranes and interfaces. To exploit the light‐triggered reactivity of photobases within such materials, we first investigated how the ground‐ and excited‐state properties of the quinoline unit changes upon polymer integration. To address this matter, we combined absorption and emission spectroscopy with time‐resolved transient‐absorption studies to reveal photoinduced proton‐transfer dynamics in various solvents. The results yield important insights into the thermodynamic and kinetic properties of these polymeric quinoline photobases.

## Graphical abstract

**Back to base**: We present the synthesis of the first polymeric photobase prepared by RAFT polymerization carrying quinolines moieties pendant to the polymer backbone. UV‐light‐induced proton capture is investigated within a representative molecular and macromolecular quinoline photobase regarding the influence on ground and excited state and enables insights into thermodynamic and kinetic properties.

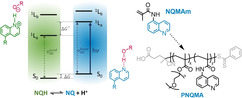

## Introduction

The control of proton‐transfer reactions by external stimuli is of interest as a tool to address chemical reactions^[1]^ and biochemical processes.^[2]^ One way to temporally and spatially control the release of protons is the use of photoacids; molecular structures that experience a drop in p*K*_a_ of several orders of magnitude upon UV light absorption.

A complementary approach to the widespread use of photoacids is to use photobases. Photobases exhibit a drastic increase in basicity in the electronically excited state, that is, p*K*_a_<p*K*_a_*. Typical representatives of photobases are aromatic amine derivatives such as quinolines,^[3]^ acridines,^[4]^ 3‐styrylpyridines^[5]^ and azomethines,^[6]^ as well as other derivatives like xanthones^[7]^ and curcumin.^[8]^ Despite the numerous studies of photoacids, particularly naphthols^[9]^ and pyrenols,^[10]^ there are only a few papers that explore the ultrafast proton‐capture dynamics of quinoline photobases: for this reason Dawlaty et al. investigated a series of substituted quinolines.[^11^, ^12^] Their research indicated that substitution in the 5‐position, and the role of triplet states contribute to the proton capture dynamics.^[11]^ Furthermore, Dawlaty established a free‐energy relation between the proton‐capture rate of 5‐methoxyquinoline in different protic solvents and the thermodynamic driving force of the protonation reaction.^[12]^ Thus far, only a few contributions have examined the integration of photoacids or photobases into polymeric materials despite the fact that the integration of p*K*_a_ switches into macromolecules opens up ways to translate light‐triggered proton release and capture to nanoparticles, membranes and surfaces. Ardo and co‐workers created a pyrenol‐photoacid‐modified Nafion ion‐exchange membrane for light‐driven ion transport used in photovoltaics.^[13]^ Furthermore, they recently studied interfacial and nanoconfinement effects on a polymer‐bound photoacid and found that nanoconfinement alters the ground‐ and excited‐state acidity, which were related to changes in the surface potential and the solvation environment.^[14]^ We previously investigated and reported the excited‐state proton transfer in a series of 1‐naphthol‐derived polymeric photoacids.^[15]^ Herein, we present the synthesis of the first polymeric photobase derived from a polymerizable quinoline derivative (*N*‐(quinolin‐5‐yl)methacrylamide, NQMAm). A representative copolymer containing NQMAm and oligo(ethylene glycol) methacrylate (OEGMA) denoted PNQMA was synthesized by RAFT copolymerization. Spectroscopic studies revealed that the macromolecular environment causes changes in the quinoline photophysics. To this end, we studied the impact of the proton source on the energetics of ground and excited states and discuss the ultrafast reaction kinetics of photoinduced proton capture, comparing the molecular photobase NQMAm to the macromolecular photobase PNQMA.

## Results and Discussion

**Figure 1:**
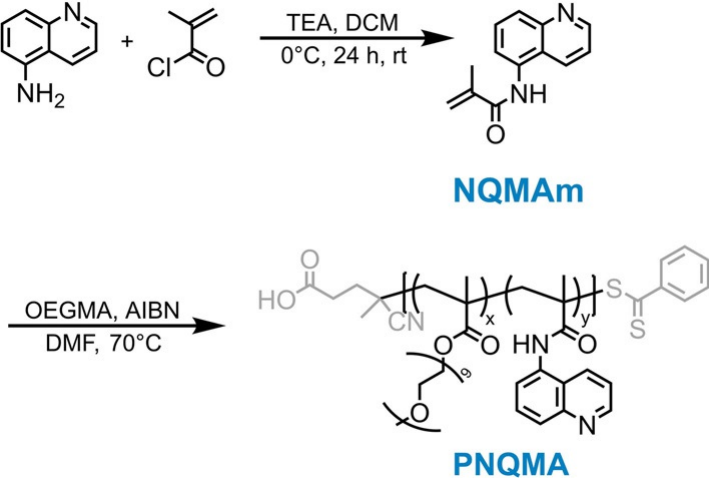
Synthetic scheme showing the synthesis of *N*‐(quinolin‐5‐yl)methacrylamide (NQMAm) and the corresponding copolymer PNQMA (*x=*0.75, *y=*0.25).

The polymerizable photobase *N*‐(quinolin‐5‐yl)methacrylamide (NQMAm) was accessed *via* a simple acylation of quinolin‐5‐amine with methacryloyl chloride using triethylamine as a base in dichloromethane (DCM) at 0 °C (Figure 1). Pure NQMAm was isolated as a white powder by column chromatography with a yield of 69 %. Characterization by ^1^H and ^13^C NMR is shown in Figures S1 and S2 in the Supporting Information.

To study the photophysical properties of a photobase in a macromolecular environment, a water‐soluble copolymer of NQMAm and OEGMA was prepared using reversible addition‐fragmentation chain‐transfer (RAFT) polymerization as depicted in Figure 1, denoted PNQMA. Under these optimized polymerization conditions, we obtained a well‐defined copolymer with a number averaged molecular weight of 11 000 g mol^−1^ and a low dispersity of 1.18. The monomer feed ratio (NQMAm/OEGMA=25:75) was in close agreement to the final polymer composition determined by ^1^H NMR analysis, indicating that a random copolymer of NQMAm and OEGMA was formed.

### Thermodynamic considerations regarding the photobasicity of NQMAm and PNQMA

**Figure 2:**
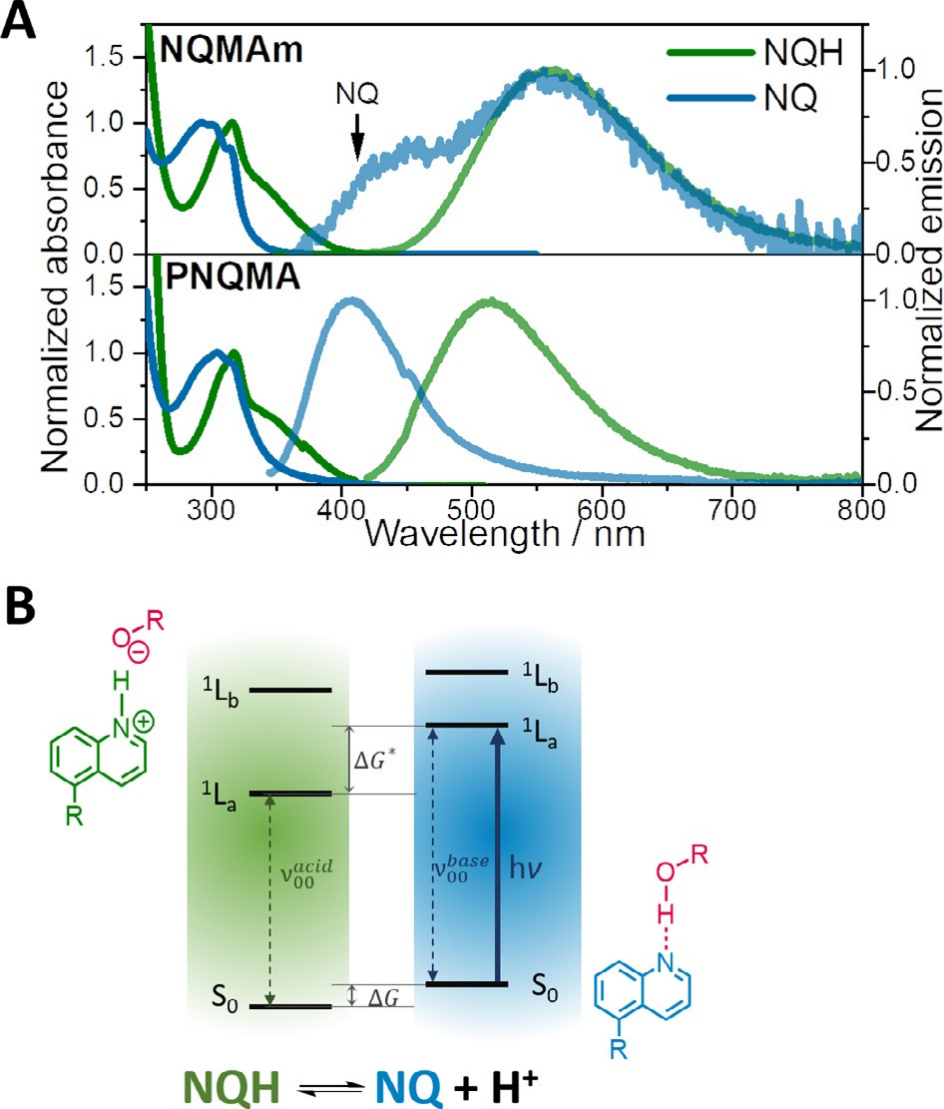
A) Ground‐state absorption and emission spectra of the quinoline and quinolinium forms, NQ and NQH, of the molecular photobase NQMAm and the quinoline functionalized polymer PNQMA. B) Thermodynamic cycle illustrating the photobasicity of quinolines. Upon electronic excitation, the quinoline photobase NQ captures a proton from a proton source with a suitable p*K*_a_, for example, alcohols or water, and forms the protonated species NQH in the excited state.

Figure 2 displays the ground‐state absorption and emission spectra of the unprotonated and protonated forms of NQMAm and PNQMA. Two electronic states contribute to the main absorption band of quinolines in the UV range, which (according to the Platt notation of cata‐condensed aromatic systems) are referred to ^1^L_a_ and ^1^L_b_ states.^[16]^ The ^1^L_a/_^1^L_b_ states are characterized by atom centered/ bond centered excess charge density in the excited state compared to the electronic ground state. Excitation of the ^1^L_a_ state pushes electron density towards the heterocyclic nitrogen and thus plays a crucial role in quinolines acting as a photobase (Figure 2).

Integration of the quinoline into the OEGMA‐based copolymer causes a bathochromic shift of the overall absorption both in the NQ and the NQH form of the photobases (see also Figure S5 and Table 1). Quantitative analysis by Gaussian fitting indicates a shift to lower energies of Δ*E*≈−0.13 eV in NQ and −0.12 eV in NQH of PNQMA with respect to the monomer. The ground‐state acidity constants p*K*_a_ were estimated via UV/Vis titration to 4.0 and 2.8 in NQMAm and PNQMA, respectively (Figures S3 and S4). The emission of the protonated quinoline NQH obtained in acidified aqueous solutions is centered at 558 nm in NQMAm and 512 nm in PNQMA. The blueshifted emission located at 405 nm in PNQMA in a basic aqueous environment corresponds to the unprotonated form NQ. The exclusive emission of the NQ species was not detected in NQMAm, which is attributed to emission quenching effects at high pH values. In neutral aqueous solution the monomeric photobase NQMAm shows dual emission, and the peak located at higher energies is assigned to the emission of the unprotonated form NQ. The corresponding energy of NQ in NQMAm was extracted by Gaussian fitting of the emission spectrum (Figure S6) and estimated to be 2.88 eV (431 nm). With this information in hand, the corresponding acidity constants in the excited state, p*K*_a_*, are derived by Förster cycle analysis^[17]^ (see depiction for quinolines in Figure 2 B, and further details in the Supporting Information page 4 and Figure S6). Both photobases NQMAm and PNQMA reveal a drastic jump in the basicity or Δp*K*_a_ of approximately 10 units upon excitation resulting in a p*K*_a_* of 14.1 in NQMAm and 12.6 in PNQMA. This indicates that integration of the quinoline motifs into the methacrylate‐based polymer does not diminish the potential of quinoline to act as a photobase.

**Table 1:** Approximated ^1^L_a_ band transition energies, emission maxima of the unprotonated and protonated form, NQ and NQH, and acidity constants in the electronic ground and excited state of NQMAm and PNQMA.

	S_0_‐^1^L_a_ NQ	S_0_‐^1^L_a_NQH	*E*_em_NQ	*E*_em_NQH	Δp*K*_a_	p*K*_a_	p*K*_a_’’
NQMAm	4.20	3.67	2.88	2.22	10.1	4.0	14.1
PNQMA	4.07	3.55	3.06	2.42	9.8	2.8	12.6

**Figure 3:**
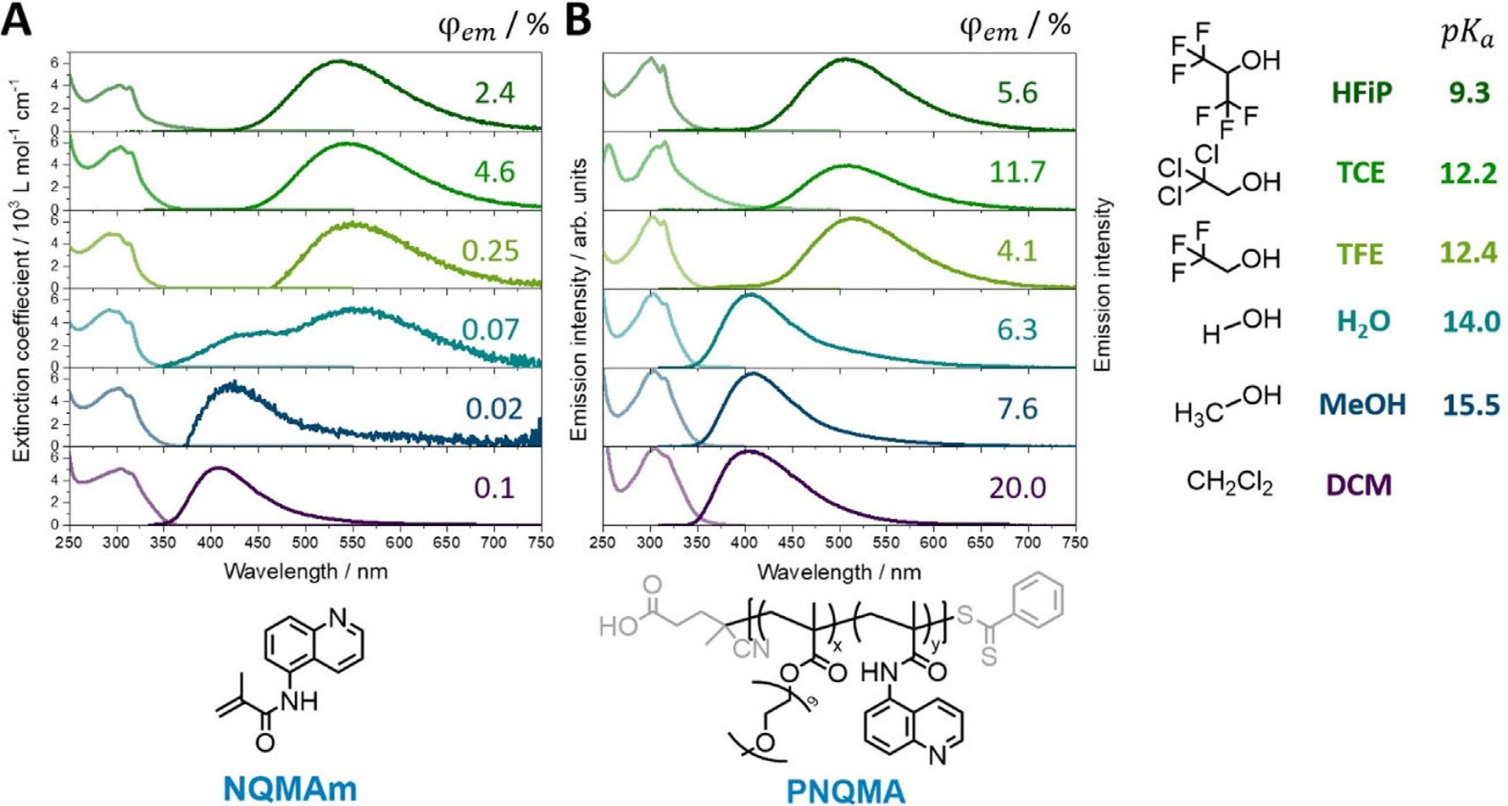
A) Absorption and normalized emission spectra of NQMAm, and B) excitation and emission spectra of PNQMA collected in protic solvents, for example, water and alcohols, as well as the aprotic solvent dichloromethane.

Variation of the solvent confirms the proton capture ability of the polymeric photobase PNQMA and compares the key spectroscopic features to the monomeric NQMAm (Figure 3 and Table S2). Both NQMAm and PNQMA exhibit ground‐state p*K*_a_ values far below the solvent p*K*_a_ (i.e., 4.0 and 2.8, see discussion above), which ensures the presence of the unprotonated quinoline NQ in the electronic ground state, while depending on the solvent excited‐state protonation is observed. In aprotic DCM, the unprotonated species NQ is present both in the ground and the excited state as indicated by the NQ emission at approximately 410 nm. In low p*K*_a_ protonating solvents—such as HFiP, TCE and TFE—the emission is distinctly redshifted compared to the NQ form, confirming the presence of the protonated quinolinium form NQH in the excited state. In H_2_O, NQMAm shows a dual emission with estimated peak positions at 431 and 558 nm, which indicates proton transfer from the solvent to a fraction of excited photobase chromophores and hence the presence of both species, that is, NQ and NQH, in the electronically excited state. These observations are in line with the estimated p*K*_a_^*^ values of NQMAm and PNQMA (Table 1), and the thermodynamic notion that proton capture to the electronically excited photobase takes place in solvents of lower p*K*_a_ compared to the p*K*_a_^*^ of the photobase. Thus, the excited‐state protonation of the quinoline photobase is not limited by its macromolecular environment, for example, due to insufficient solvation of the chromophore and the generated photoproducts in the respective polymer‐solvent environment. However, the solvent also affects the energetics of the ground and the excited states. To illustrate this, we compare the absorption and emission features of NQMAm and PNQMA in TFE and DCM, a protic and an aprotic solvent of similar polarity (Table S2, *ϵ*_r_=8.6 and 8.9 F m^−1^). Thus, changes observed within absorbance and emission are directly relatable to changes in the protonation state of the quinoline and account for pre‐association, that is, the formation of hydrogen bonded photobase associates, NQ HO‐R, in proton donating solvent. Upon addition of TFE to a solution of the photobase in DCM the main absorption band gradually blueshifts (Figure S7A). This agrees with the absorption peak difference in TFE and DCM, which amounts to +0.42 eV and +0.16 eV in NQMAm and PNQMA, respectively. The blueshift of the absorption band observed upon addition of the hydrogen bond donor TFE indicates a destabilization of an excited state exhibiting n–π* character.^[18]^ The opposite effect is observed in the excited state: addition of a proton source TFE (ca. 2000 equiv. based on the amount of NQ) redshifts the emission band by −0.10 eV and −0.11 eV in NQMAm and PNQMA (Figure S9). Such a shift to higher energies upon the presence of small molar fractions of a proton donor is related to the stabilization of a H‐bonded π–π* state, and interpreted as a strengthening of the hydrogen bond in the excited state.^[19]^ This strengthening effect of the ES hydrogen‐bond complex NQ**⋅⋅⋅**HO−R correlates with the enhanced basicity in the excited state. In general, the absorbance and the emission spectra in the considered solvent set are less effected by solvent polarity and proton donor capability within the macromolecular photobase PNQMA (Figures S7 and 8, Table S1). Negative solvatochromism, which manifests in a redshift in the emission obtained in non‐protonating solvents of increasing polarity, as seen in NQMAm, is not distinguishable in PNQMA (Figure S7C). This indicates that the major environmental effect within PNQMA is introduced by oligoethylene glycol in the copolymer side chains. The emission quantum yield of NQMAm is dependent on the protonation state of the quinoline. In solvents with p*K*_a_ values that mediate protonation of NQMAm in the excited‐state moderate fluorescence quantum yields, that is, in the range of a few percent, are observed (Table S3). In higher p*K*_a_ solvents, in which no excited‐state proton transfer (ESPT) takes place, the emission quantum yield of NQ is reduced (e.g., 2.4 % in HFiP compared to <0.1 % in MeOH). In general, the emission quantum yield increased upon integration of the quinoline into the copolymer and was found to be independent of the protonation state. In all solvents under investigation PNQMA revealed emission quantum yields in the range of 5–20 % (Figure 3). *N*‐Heterocyclic compounds reveal closely spaced n‐π* and π–π* transitions. As reported in literature, low emission quantum yields of azarenes, for example, quinolines in their respective unprotonated form NQ, can be related to the lowest‐lying excited singlet state with n–π* character which decays dominantly *via* non‐radiative decay channels.^[18]^ However, the nature of the two lowest singlet states is strongly affected by inter‐ and intramolecular polar interactions, for example, by the specific solvent environment and the microenvironment created by the (macro‐)molecular structure. The polar interactions (and the presence of hydrogen bond donors) tend to destabilize the n‐π* transition whereas the π–π* transition is stabilized. This can lead to an inversion^[18]^ or to state mixing of the two lowest electronic states.^[20]^ The second situation, that is, an emissive state with partial π–π* character, may explain the higher emission quantum yield of PNQMA in its unprotonated form NQ.

### Photoinduced proton capture dynamics of NQMAm and PNQMA in various solvents

The excited‐state dynamics of the quinoline photobases were investigated in various solvents, that is, HFiP, TCE, TFE, H_2_O, MeOH and DCM, were investigated by femtosecond transient‐absorption (TA) spectroscopy. This study gives insights into the i) role of the different solvents and the ii) impact of polymer integration on the proton capture dynamics of the photobase.

### fs‐TA spectroscopy in solvents not allowing for excited‐state protonation of NQ

The transient spectra collected in the aprotic solvent DCM (Figure S13) reveal a broad positive differential signal covering the entire probe wavelength range accessible. The initial spectrum (Δ*t*=0.5 ps) shows minor structuring with a maximum at 435 nm in both chromophores and an additional band located at 380 nm in NQMAm. At later delay times a hypsochromic shift of the band from 435 nm to 420 nm is followed by the overall decay of the transient signal. The collected data was analyzed by a multi‐exponential fit to estimate the corresponding characteristic first order time constants (Table S3). The initial decay associated with the blueshift ESA band at around 435 nm is characterized by *τ*_1_ (2 ps in NQMAm and 33 ps in PNQMA). This kinetic component is interpreted as solvent relaxation of the photoexcited chromophore.^[21]^ Subsequently, the unprotonated quinoline returns to the electronic ground state on a *ns* timescale, that is, with a characteristic time‐constant of approx. 3.1 ns in PNQMA and 2.6 ns in NQMAm. Also, the obtained time constants *τ*_1_=32 ps and *τ*_2_=2.3 ns for PNQMA in MeOH resemble the results in DCM. For NQMAm in MeOH a fast time constant of *τ*_1_=1.8 ps is observed, which can be assigned to the decay of a red‐shifted excited‐state absorption feature, yielding an ESA band below 400 nm, which decays with a characteristic time constant of 20 ps. Hence, *τ*_1_ is associated again with cooling, while we conclude that the decay of the excited‐state monomeric photobase in MeOH (20 ps) is drastically faster than in its polymeric counterpart (2.3 ns). We associate this process with the presence of fluctuating hydrogen bonds between the NQ units and MeOH in NQMAm and PNQMA. Nonetheless, the solvent‐independent spectral signatures of the photoinduced dynamics in NQMAm and in PNQMA in DCM and MeOH confirm the absence of excited‐state proton transfer. This result agrees with the estimated insufficient thermodynamic driving force of Δp*K*_a_ −1.4 in NQMAm and −2.9 in PNQMA for excited‐state protonation.

### fs‐TA spectroscopy in solvents allowing for excited‐state protonation of NQ

**Figure 4:**
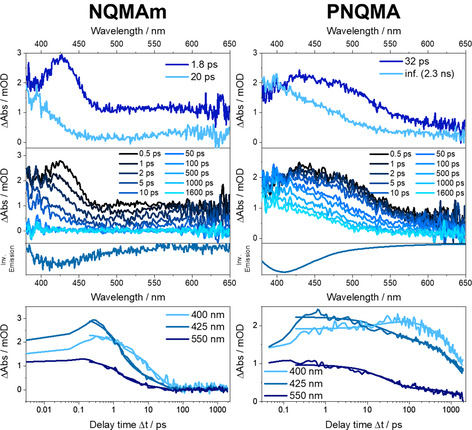
Compilation of fs‐transient absorption data of NQMAm (left) and PNQMA (right) obtained in the solvent MeOH (p*K*_a_=15.5, no ES protonation of NQ). Upper panels of the graphs show the species‐associated difference spectra (SADS) and transient spectra obtained at selected delay times Δ*t* after photoexcitation together with an inverted emission spectrum. The lower panels include kinetic traces at selected wavelengths.

**Figure 5:**
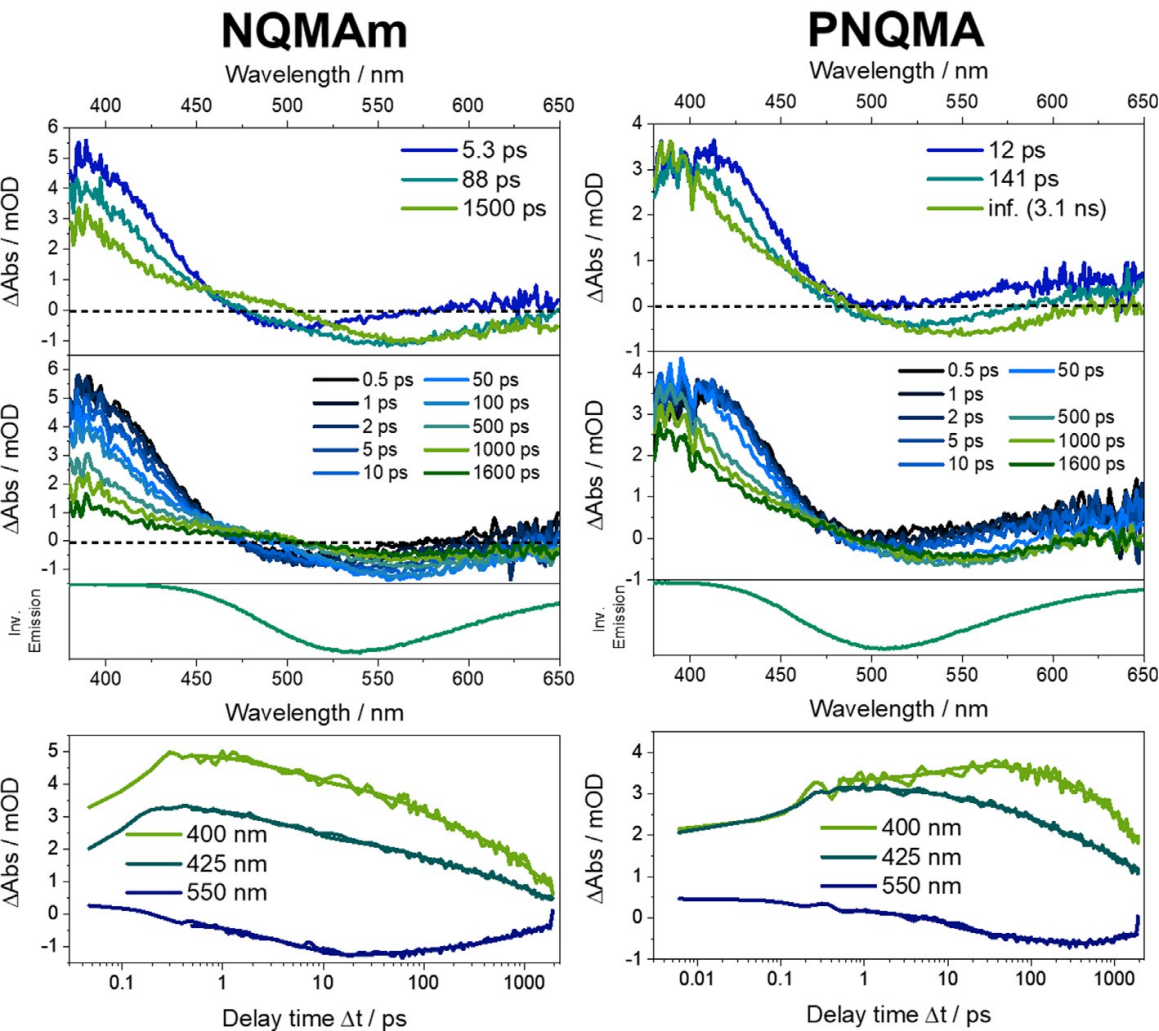
Compilation of fs‐transient absorption data of NQMAm (left) and PNQMA (right) obtained in the solvent HFiP (p*K*_a_=9.3, ES state protonation of NQ). Upper panels of the graphs show the SADS and transient spectra obtained at selected delay times Δ*t* after photoexcitation together with an inverted emission spectrum. The lower panels include kinetic traces at selected wavelengths.

When dissolved in HFiP, the transient absorption spectrum of NQMAm recorded at Δ*t*=0.5 ps is dominated by a positive differential absorption signal below 450 nm and beyond 600 nm corresponding to ESA in these spectral regions (Figure 5). At around 510 nm, minor negative contributions are visible, which we relate to the stimulated emission in this spectral region. At increasing delay times, the features below 450 nm decreases, whereas the negative band grows and shifts to longer wavelengths. From delay times of about 5 ps onward, an isosbestic point at 452 nm is distinguishable. At longer delay times, the negative band appears at 560 nm and an overall decay of the transient absorption feature is observed. This decay is incomplete within the experimentally accessible delay time window. The fs‐TA spectra of PNQMA dissolved in HFiP show qualitatively the same features as NQMAm. Furthermore, dissolving NQMAm and PNQMA in TCE and TFE yields qualitatively identical results as in HFiP (Figures S10 and S11). The quantitative data analysis is based on a global three‐exponential fit, and the resulting characteristic time constants are summarized in Table S3. Based on the transient absorption spectra collected in non‐protonating solvents, that is, MeOH and DCM (Figure 4 and Supporting Information Figure S13), the differential absorption spectra obtained immediately after photoexcitation are related to the unprotonated quinoline NQ. However, the presence of a stimulated emission band located at approximately 510 nm indicates that partial quinoline proton capture already takes place within the first 0.5 ps after photoexcitation (see also Figure S16). We propose that the slight changes in the transient spectra in early times are related to excited‐state solvation and reorganization of the hydrogen‐bonded quinoline photobase, that is, the formation of an intermediate protonated state exhibiting a strengthened hydrogen‐bond between quinoline and the proton donating solvent. The timescale of solvation for the molecule agrees with solvation times available in literature.^[22]^

**Figure 6:**
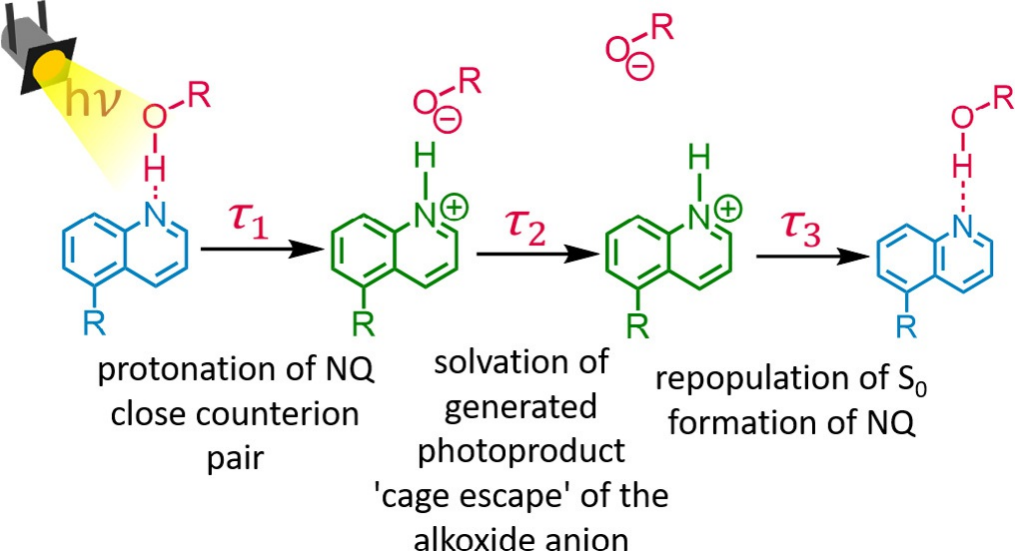
Depiction of the ES dynamics observed in the quinoline photobases NQMAm and PNQMA.

The process associated with *τ*_1_ (5.3 ps in NQMAm and 12 ps in PNQMA, HFiP, Figure 5) reflects the decrease of the ESA band at around 400 nm and further build‐up of the SE which simultaneously red shifts from 510 to 540 nm in NQMAm and to 525 nm in PNQMA, respectively. Within the observation window, for example, from 5 ps onwards (NQMAm in HFiP), an isosbestic point at 452 nm emerges. This indicates a population transfer between two distinct chemical species, that is, NQ and NQH. For that reason, we assign the process associated with *τ*_1_ to excited‐state proton capture by the quinoline photobase and the formation of a closely interacting counterion pair (Figure 6). Integration of the photobases into a copolymer slows down the kinetics for proton capture (see also Figures S14 and S15). The respective characteristic first‐order time constant *τ*_1_ is 5 ps for NQMAm in HFIP, while it is prolonged to 12 ps for PNQMA. In parts, the slower proton capture in a macromolecular environment can be related to a reduced thermodynamic driving force: the p*K*_a_* of PNQMA is 1.5 units lower compared to the respective molecular photobase NQMAm. Furthermore, changes in the local solvation and correspondingly different local dielectric constants imposed by the polymer side chains might contribute to the kinetic differences observed. The second kinetic component, *τ*_2_, features a decrease of the ESA band located at around 400 nm and the emergence of a stimulated emission located at 565 nm. We assign this process to “anion escape” from the local solvent cage, that is, the separation of the protonated photobase and the acid anion. Such anion escape has not been observed in ultrafast proton transfer studies involving quinoline photobases before, likely due to the limited delay‐time window of 600 ps employed in studies so far.[^11^, ^12^, ^23^] Nevertheless, a sequential proton transfer was previously identified during ESPT of a quinone cyanine 9 photoacid^[24]^ and a Schiff base super‐photobase.^[6]^ The anion escape is prolonged in the polymeric photobase, for example, characterized by a time constant of 90 ps in NQMAm compared to approx. 140 ps in PNQMA considering HFiP as solvent. The obtained results in all protonating solvents under investigation show that also the generation of fully charge separated quinoline photobases is notably slower in the macromolecular environment. *τ*_3_, which is characterized by the decay of the ESA band at 400 nm accompanied the vanishing of the SE band is interpreted as repopulation of the electronic ground state by NQH followed by the (re‐)formation of the unprotonated quinoline NQ.

In summary, the implementation of a photobasic quinoline into a copolymer environment does not affect the proton capture ability itself but has drastic effects on the overall dynamics observed upon photoexcitation of the chromophore. A simplified scheme of the ES dynamics in quinolines is depicted in Figure 6 (representative for the PNQMA, but also valid for the molecular photobase NQMAm). Three different states were distinguished along the proton capture of the quinoline: i) solvation and hydrogen bond strengthening; ii) excited‐state protonation, and thus the formation of a close counter ion pair; and iii) “cage escape” of the alkoxide anion, that is, formation of completely separated photoproducts. All processes proceeded slower in a macromolecular environment with polar oligoethylene glycol side chains.

## Conclusions

We report the integration of quinoline photobases in a methacrylamide copolymer as a proof‐of‐concept example of a photoswitchable polymer material. Analysis of the ground‐state absorption spectra shows that embedding the quinoline affects the energy levels of the ^1^L_a_ transitions, as manifested in a bathochromic shift of approximately 0.12 eV. Nevertheless, the change in p*K*_a_ upon UV excitation was estimated to be approximately 10 units in both quinoline chromophores, yielding an excited state of 14.1 in NQMAm and 12.6 in PNQMA. Our contribution demonstrates that the local dielectric constant has a major impact on the photophysical properties of quinoline photobases. We further demonstrated that the polymer environment greatly influences the observed excited‐state proton‐capture kinetics, for example, slowing down proton capture of the quinoline from solvents, which is attributed to i) a lowered thermodynamic driving force and ii) the increasing impact of the local dielectric constant introduced by the oligo(ethylene glycol) in the polymer side chain. The rather long‐lived excited state of the macromolecular quinoline photobase under investigation seems to be very beneficial for applications that require the removal of a proton, for example, in photocatalysis. While this study has shown the general ability of quinolines acting as proton‐capture units in polymers, we still face great challenges to design intelligent and light‐switchable soft matter materials based on photobases.

## Experimental Section

5‐Aminoquinoline (>99 %) was purchased from TCI Deutschland GmbH. Methacryloyl chloride (97 %) was purchased from Alfa Aesar. Triethylamine (99.0 %) was purchased from Chem Solute. Dichloromethane was purified using a PureSolv‐EN solvent purification system (Innovative Technology). Oligo(ethylene glycol)methyl ether methacrylate (OEGMA, *M*_n_=500 g mol^−1^) was purchased from Sigma–Aldrich and passed over a short column of inhibitor remover packing (Sigma–Aldrich) to remove the inhibitor prior to use. 2,2’‐Azobis(iso‐butyronitrile) (AIBN, Sigma–Aldrich) was recrystallized from ethanol and stored in a freezer until use. 4‐Cyanopentanoic acid dithiobenzoate (CPADB, ≥97.0 %) was obtained from Strem Chemicals and used as received. *N*,*N*‐Dimethylformamide (DMF, peptide grade) was purchased from Acros Organics. All other chemicals and solvents were purchased from Sigma–Aldrich and used without further purification unless stated otherwise.

### Nuclear magnetic resonance

NMR spectroscopy was performed on a 300 MHz Bruker AC spectrometer at 298 K with the residual solvent resonance as an internal standard. The chemical shifts on the *δ* scale are given in ppm.

### Size‐exclusion chromatography

SEC was carried out on an Agilent 1200 series system equipped with a G1310A pump, a G1315D DA detector, a G1362A RI detector, and with PSS GRAM 30 Å/1000 Å (10 μm particle size, Polymer Standards Service GmbH, Mainz, Germany) columns in series at 40 °C using *N*,*N*‐dimethylacetamide (DMAc) with 2.1 g L^−1^ LiCl as eluent at a flow rate of 1 mL min^−1^. The system was calibrated with PMMA standards (*M*_p_=505 to 981 000 g mol^−1^).

### Synthesis of *N*‐(quinolin‐5‐yl)methacrylamide (NQMAm)^[25]^

Quinolin‐5‐amine (2.5 g, 17.3 mmol) was weighed out into a 100 mL round‐bottom flask charged with a magnetic stirrer bar. The flask was then sealed with a rubber septum before being evacuated and back‐filled with argon 3 times. Dichloromethane (DCM, 60 mL) was then added to dissolve the quinolin‐5‐amine. The mixture was then cooled in an ice bath to 0 °C under vigorous stirring before triethylamine (3.6 mL) was added via syringe. Methacryloyl chloride (1.51 mL) was then added dropwise to the solution via syringe. After 30 min, the ice bath was removed, and the reaction was allowed to proceed for a further 23 h at ambient temperature before being diluted with DCM. The solution was then transferred to a separating funnel and extracted with saturated aqueous NaHCO_3_, saturated brine, and deionized water (3 times each). The aqueous phase was then further extracted with DCM before the organic phases were combined and dried over Na_2_SO_4_. The organic phase was then concentrated under reduced pressure to yield the crude product as a brown crystalline solid. The final product, *N*‐(quinolin‐5‐yl)methacrylamide, was obtained by column chromatography (eluent cyclohexane/EtOAc 1:5) as a white powder (2.54 g, yield: 69.1 %). ^1^H NMR (300 MHz, CDCl_3_): *δ*=8.92 (dd, 1 H, C_ar_‐H), 8.16 (d, 1 H, C_ar_‐H), 8.00 (d, 1 H, C_ar_‐H), 7.91 (br, NH), 7.85 (d 1 H, C_ar_‐H), 7.67 (m, 1 H, C_ar_‐H), 7.42 (dd, 1 H, C_ar_‐H), 5.94 (s, 1 H, C=C*H*), 5.56 (s, 1 H, C=C*H*), 2.14 ppm (s, 3 H,=C‐C*H*_3_); ^13^C NMR (300 MHz, CDCl_3_): *δ*=167.39 (C=O), 150.51 (*N*‐CH), 148.74 (*C=C*H_2_), 140.58 (C_ar_‐NH), 132.49 (C_ar_‐N), 130.16 (CH), 129.40 (CH), 127.86 (C_ar_‐H), 123.28 (C_ar_‐H), 122.19 (C=CH_2_), 121.17 (C_ar_‐H), 120.73 (C_ar_‐H), 19.01 ppm (CH_3_).

Synthesis of the quinoline photobase copolymer P(NQMAm_0.25_‐*co*‐OEGMA_0.75_)_9 k_ (PNQMA) by reversible addition‐fragmentation chain‐transfer (RAFT) polymerization CPADB (13.8 mg, 4.94×10^−2^ mmol), NQMAm (45.3 mg, 4.94×10^−1^ mmol) and OEGMA (0.49 mL, 1.1 mmol) were weighed out into a microwave vial charged with a magnetic stirrer bar. A stock solution containing the initiator, AIBN (4.32 mg, 2.63×10^−1^ mmol), in DMF was then added. The total volume of solvent was then adjusted to 0.46 mL to yield a DMF to OEGMA ratio of approximately 1:1 (*v*/*v*). The reaction vessel was then sealed with an aluminum cap fitted with a PTFE‐faced silicone septum, and the reaction mixture deoxygenated by purging with argon for 10 min before being placed into a thermostated oil bath preheated to 70 °C to initiate the polymerization. The polymerization was quenched after 8 h by cooling in liquid nitrogen and exposure to air. The crude mixture was diluted with DCM and precipitated in cold diethyl ether (3 times). The final copolymer was isolated by centrifugation and dried in vacuo. ^1^H NMR (300 MHz, CD_2_Cl_2_): *δ*=9.04–7.24 (quinoline), 4.42–3.96 (‐OC*H*_2_CH_2_‐(EO)_8_‐), 3.92–3.38 (‐C*H*_2_ (*EO*)_8_‐), 3.33 (‐*O*‐C*H*_3_), 2.31–0.62 ppm (backbone); SEC (DMAc/LiCl, PMMA calibration): *M*_n_=10,980 g mol^−1^, *M*_w_=12,990 g mol^−1^, *Đ*=1.18.

### Steady‐state spectroscopy

Steady‐state absorption spectra were collected either on a Jasco V530 or on a Jasco V780 UV/Vis/NIR spectrophotometer. Steady state emission spectra were collected in 1 cm quartz cuvettes, and all experiments performed on a FLS980 emission spectrometer (Edinburgh Instruments) using a Xe lamp (ozone free 450 W Xenon bulb) as an excitation source. Fluorescence quantum yields were determined relative to the reference standard 1‐napththol in H_2_O.

### Femtosecond transient absorption spectroscopy

Femtosecond transient absorption spectra were collected using a previously reported home‐built pump‐probe laser system that is based on an amplified Ti:Sapphire oscillator (Libra, Coherent Inc.).^[26]^ The excitation beam (*λ*≈340 nm, pulse duration ≈120 fs) was generated using the frequency‐doubled fundamental. The power of the pump beam was kept below 0.12 mW before entering the sample solution. A white light supercontinuum generated by an eccentrically rotating CaF_2_ plate was used to probe the photoinduced absorption changes in a wide spectral range from 350 to 750 nm. The probe beam is delayed in time with respect to the pump beam by means of an optical delay line, and the mutual polarization between the probe and pump is set to the magic angle (54.7°). The fs‐transient absorption data is displayed after a numerical chirp correction. To avoid contributions from the coherent artefact during pulse overlap,^[27]^ the region of pulse overlap was fitted by Gaussians and omitted from data analysis. Afterwards, the data was analyzed by a global multi‐exponential fit, in which the transient data below 380 nm was omitted from the data analysis due to pump‐scatter in this spectral region.

## Conflict of interest

The authors declare no conflict of interests.

## Supporting information

As a service to our authors and readers, this journal provides supporting information supplied by the authors. Such materials are peer reviewed and may be re‐organized for online delivery, but are not copy‐edited or typeset. Technical support issues arising from supporting information (other than missing files) should be addressed to the authors.

Supplementary
