# Supplementary material for: Quinoline Photobasicity: Investigation within Water‐Soluble Light‐Responsive Copolymers
Source: Chemistry. 2020 Dec 4;27(3):1072–9. doi: 10.1002/chem.202003815 (PMC7839697; doi:10.1002/chem.202003815)
Supplement: Supplementary file 1 — Supplementary [file CHEM-27-1072-s001.pdf]

Chemistry–A European Journal

Supporting Information

Quinoline Photobasicity: Investigation within Water-Soluble Light-Responsive Copolymers

Maria Sittig,[a, b, d] Jessica C. Tom,[c, d] Johanna K. Elter,[c, d] Felix H. Schacher,*[c, d] and Benjamin Dietzek*[a, b]

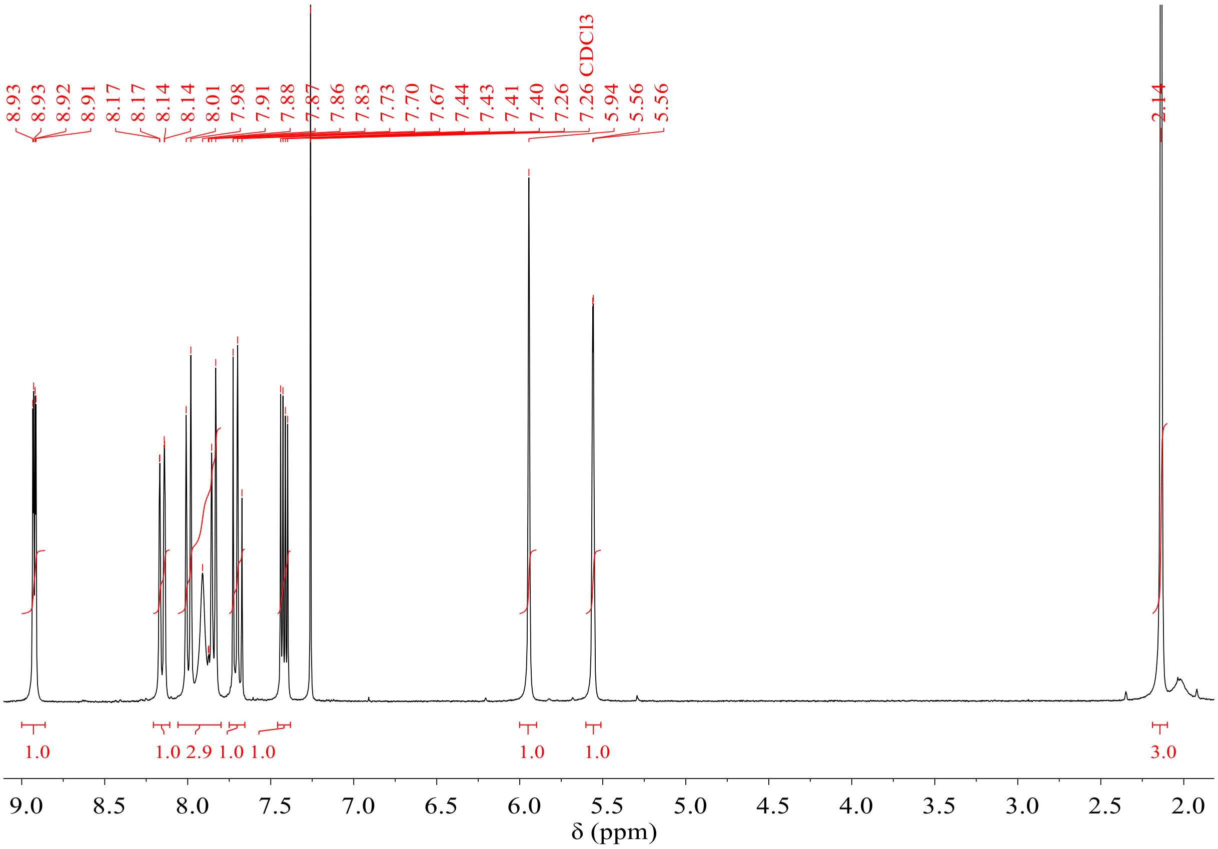

Figure S1 ^{1}H NMR spectrum of **NQMAm** in $CDCl_3$.

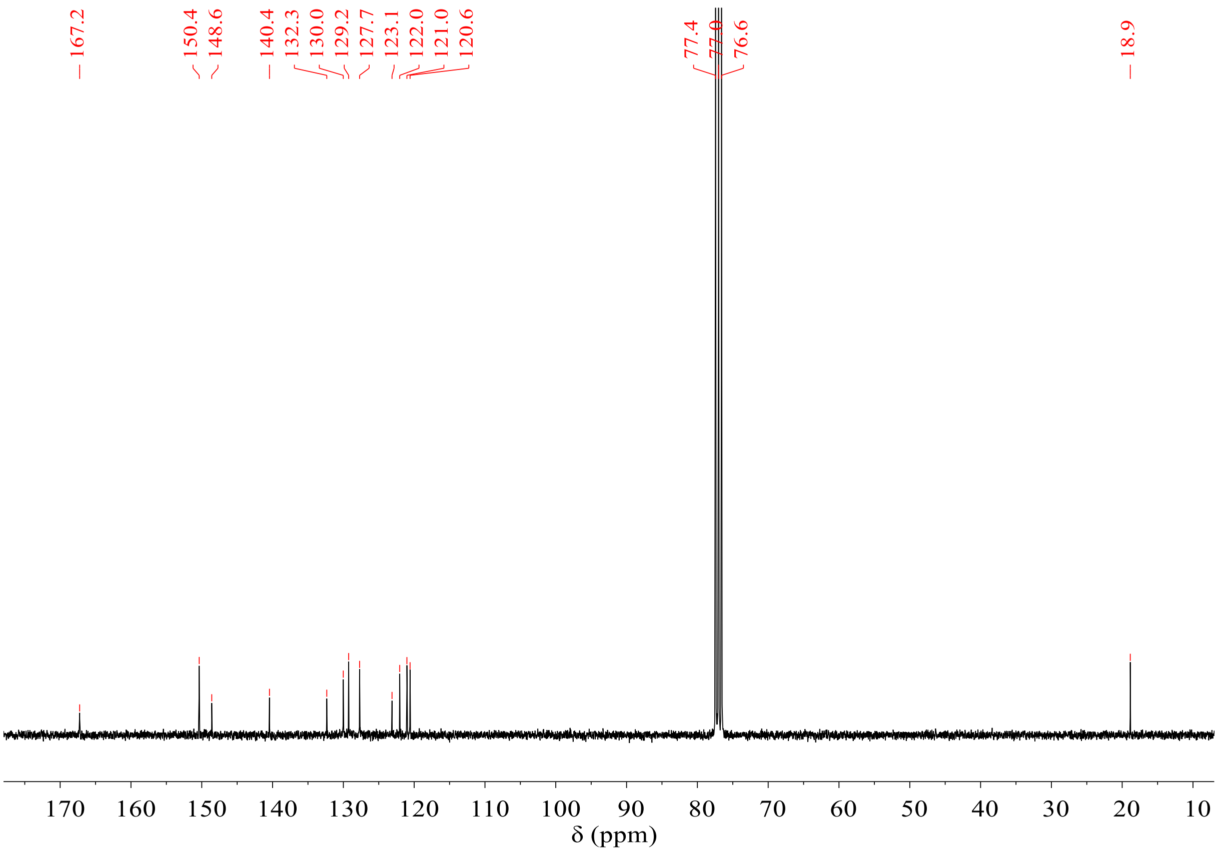

Figure S2 ^{13}C NMR spectrum of **NQMAm** in $CDCl_3$.

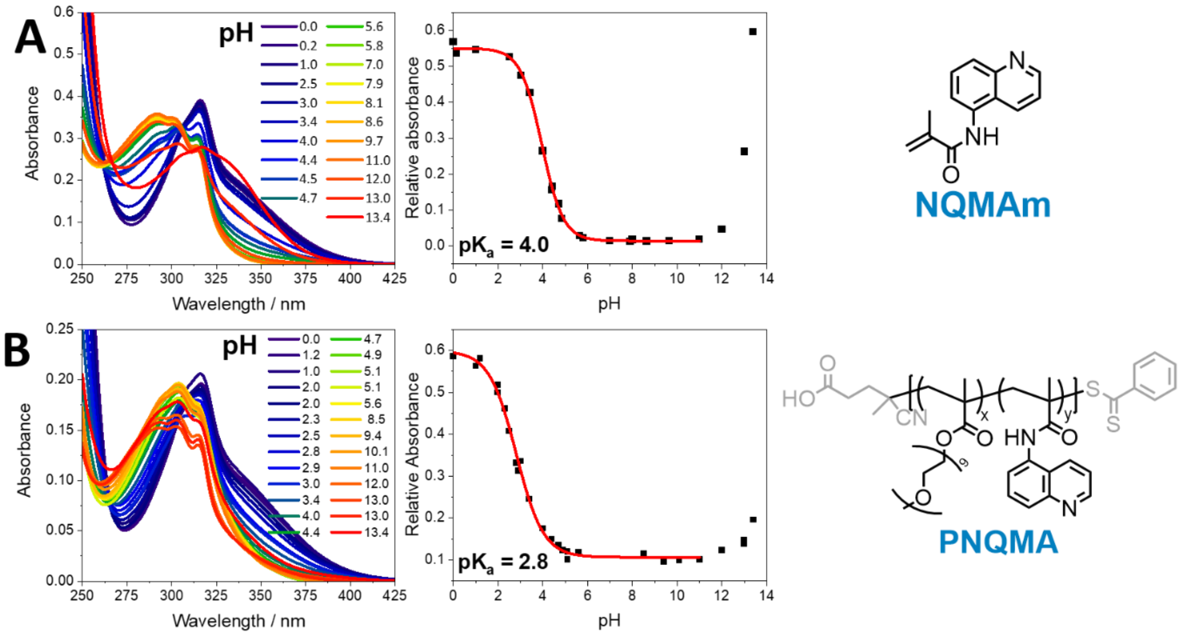

Figure S3 Absorption spectra in aqueous solution collected at different pH values (left) and titration curve (right) of (A) **NQMAm** and (B) **PNQMA**.

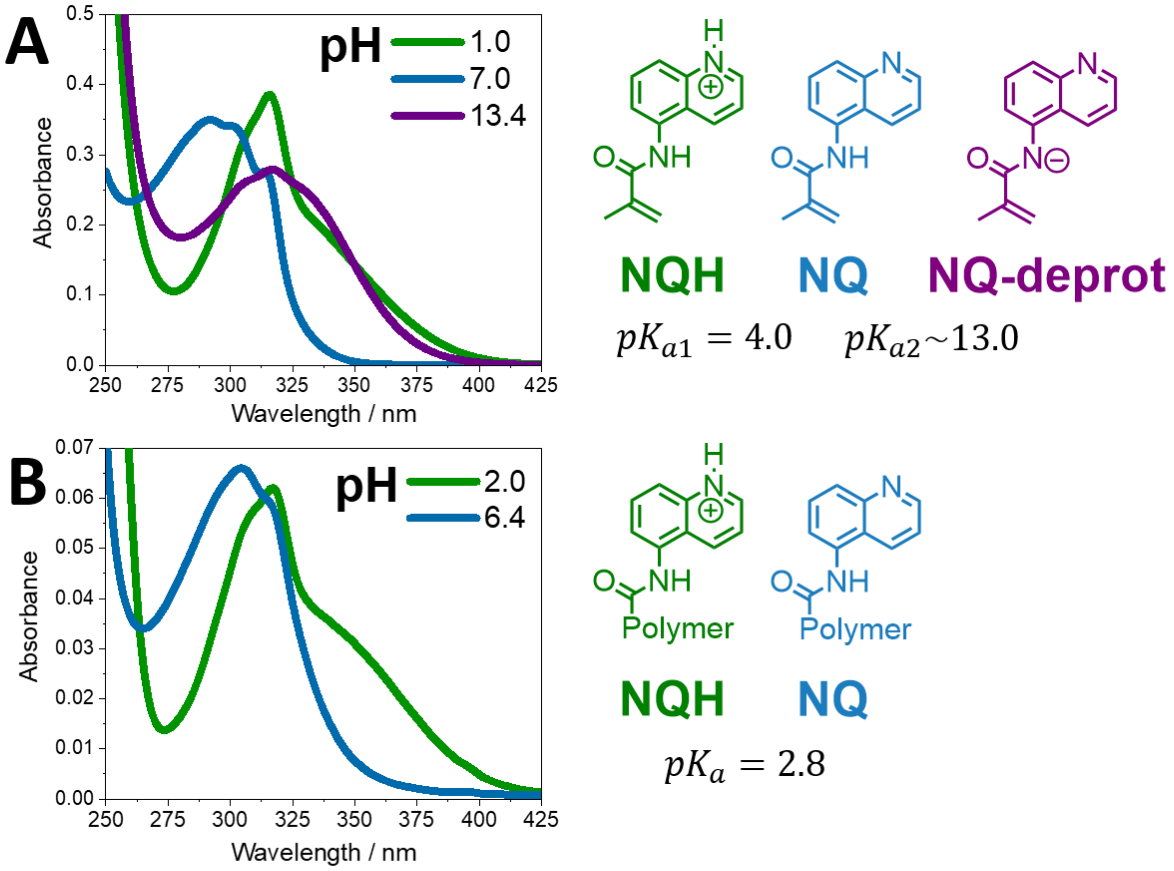

Figure S4 Absorbance spectra in aqueous solution shown at different pH values: (A) **NQMAm** and (B) **PNQMA**.

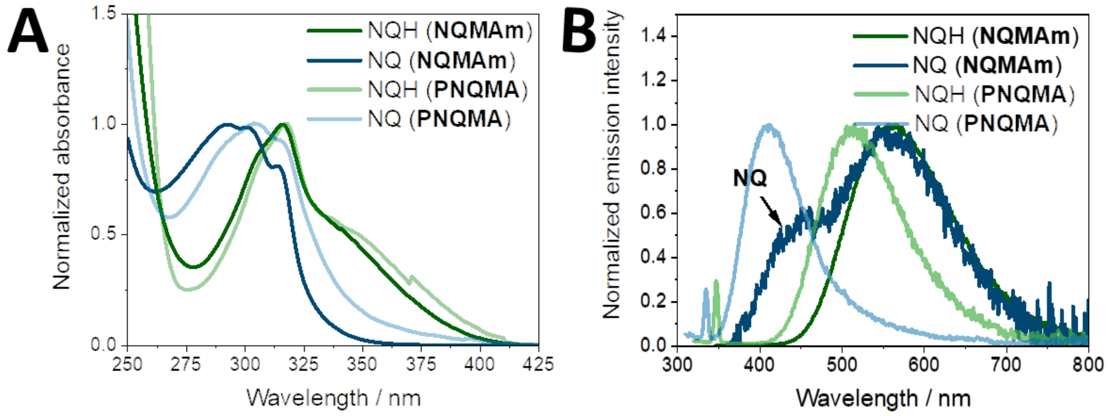

Figure S5 (A) Normalized absorbance (B) and emission spectra of NQ and NQH in **NQMAm** and **PNQMA**.

Application of the Förster cycle – Approximation of ΔpK_a^*

The thermodynamic cycle depicts the energy levels of the unprotonated and protonated form of quinoline, *i.e.* NQ (base form) and NQH (acid form), in their respective electronic ground and excited state. The ground state pK_a is accessible from the ground state energy difference of the unprotonated and protonated form ΔG, as $pK_a = \Delta G/2.3RT$. In general, the pK_a can be addressed by ground state absorption measurements and is commonly estimated *via* UV/vis titration. The 0-0 transitions of the base and acid form, v_{00}^{base} and v_{00}^{acid}, refer to the energy difference between the ground state and the lowest electronic excited state of NQ and NQH. Knowing the 0-0 transition energies of NQ and NQH and ΔG enables the estimation of the difference in the excited state energy of both species ΔG^* and the acidity constant in the excited state, as $pK_a^* = \Delta G^* /2.3RT$.

A thermodynamic consideration concerning the photobasicity in quinolines can be made by the Förster cycle,[1] which is defined by the following equation:

$$\Delta H^* - \Delta H = hc(v_{00}^{base} - v_{00}^{acid})$$

With the assumption that $\Delta S^* = \Delta S$, this equation can be directly related to the difference in the Gibb`s free energies of the electronic ground state and the respective singlet excited state:

$$\Delta H^* - \Delta H = \Delta G^* - \Delta G$$

With the following equation the Gibbs free energy can be related to an equilibrium constant, in this special case the protolysis reaction:

$$\Delta G = -k_B\, T\, lnK_a = \frac{k_B T}{log(e)}\, pK_a$$

Thus, the ΔpK_a is obtained by the following equation:

$$\Delta pK_a = \frac{\log(e)\, h\, c}{k_B\, T}(v_{00}^{base} - v_{00}^{acid})$$

The crucial 0-0 transition energies are estimated by averaging the absorption and emission maxima of both species, NQ and NQH. Prior to this, the contribution of the protonation sensitive ^1L_A state to the overall absorbance spectrum was extracted by fitting the UV/vis spectra with Gaussians (see**Figure** S6). In doing so, the lowest peak exhibiting a bathochromic shift upon protonation was used in the approximation. Assuming standard conditions, *e.g.* T = 298 K, the equation can be simplified to yield the following expression:

$$\Delta pK_a = \frac{\Delta \nu_{00}}{477\ cm^{-1}}$$

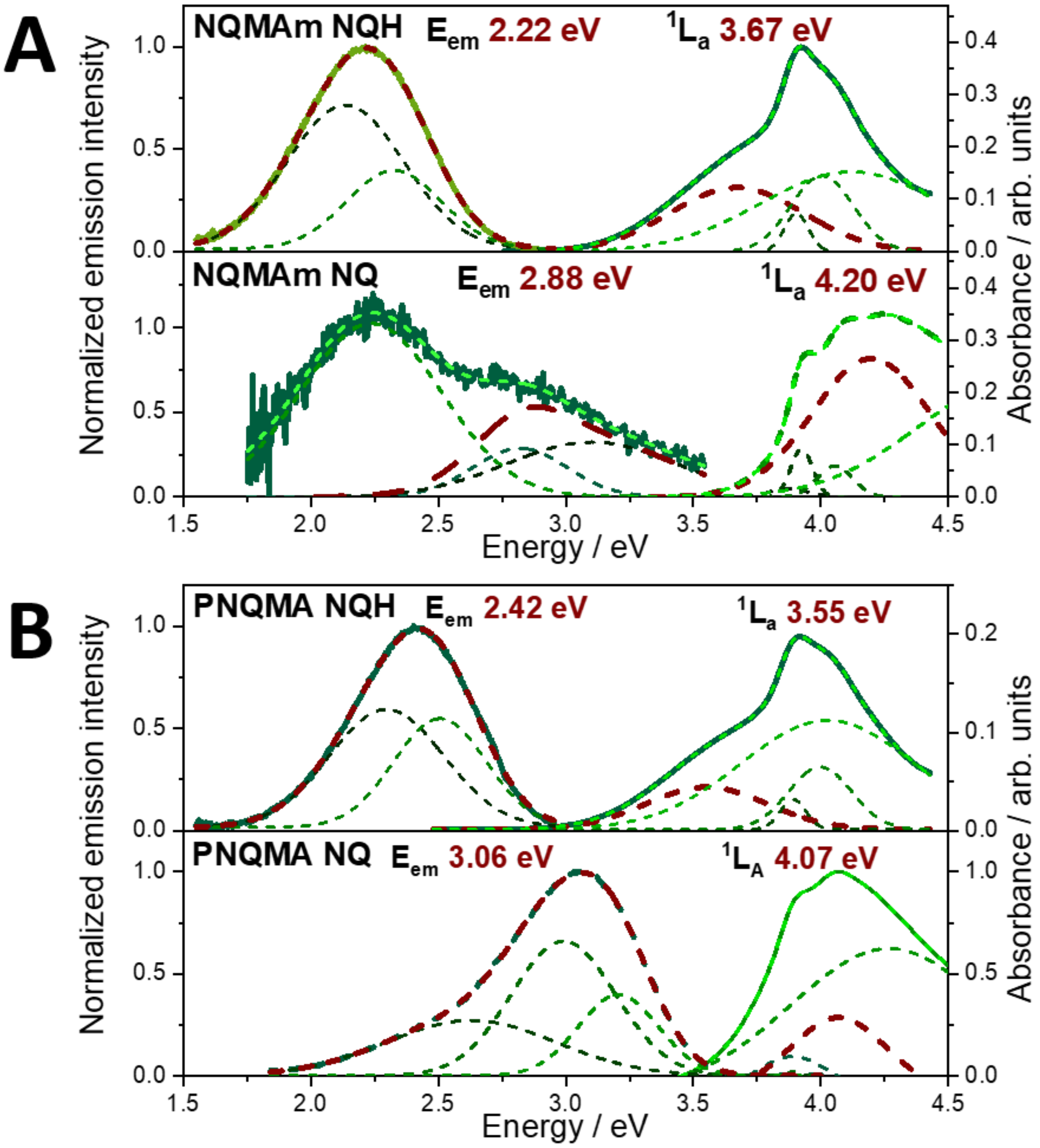

Figure S6 Depiction of the Förster cycle approximation of (A) **NQMAm** and (B) **PNQMA** in aqueous solution.

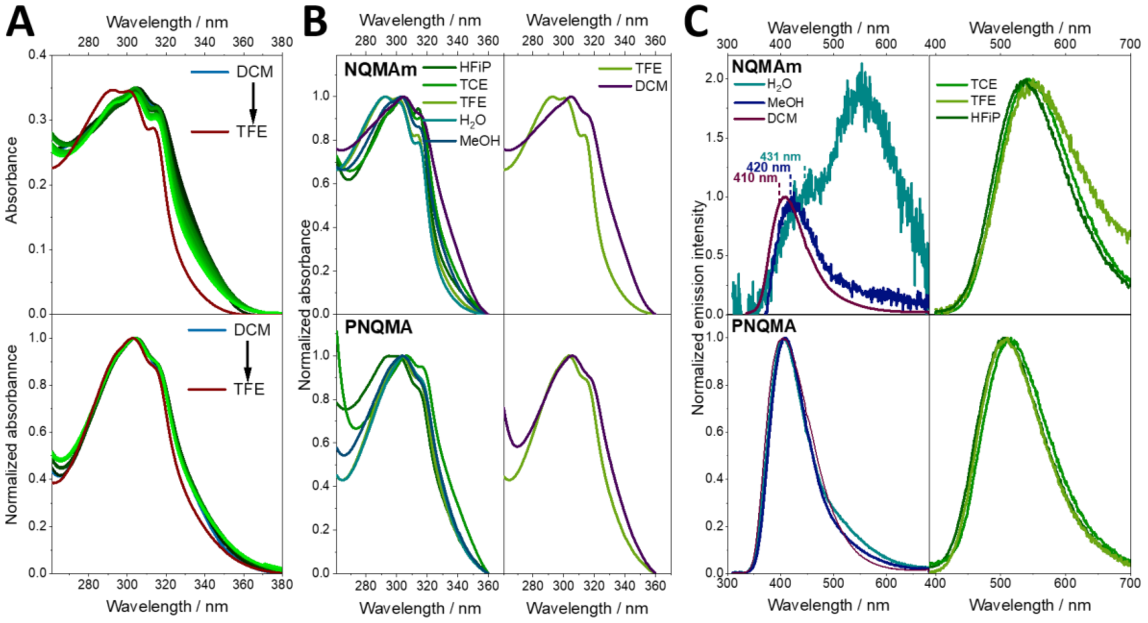

Figure S7 (A) Absorbance spectra obtained in titration experiments: solvent = DCM, titrator = TFE, titrand = **NQMAm** and **PNQMA**; (B) Normalized absorbance spectra collected in different alcohols and water, and normalized absorbance spectra collected in TFE (ε_r = 8.6 F m^{-1}) and DCM (ε_r = 8.9 F m^{-1}); and (C) Normalized emission spectra of the unprotonated quinoline NQ in H_2O, MeOH and DCM, and normalized emission spectra of the protonated quinolinium NQH in TFE, TCE and HFiP.

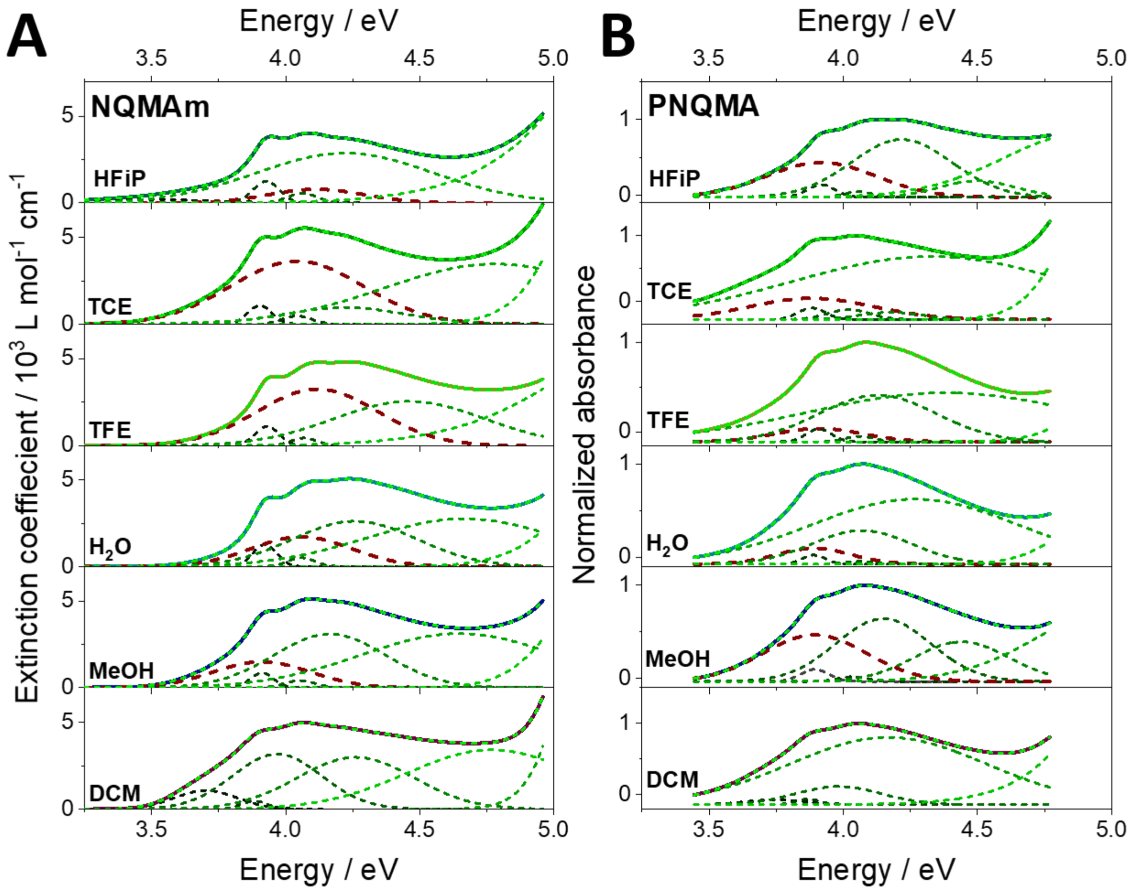

Figure S8 Ground state absorption spectra of (A) **NQMAm** and (B) **PNQMA** in various solvents fitted with Gaussians.

Table S1 Estimated energy level positions of the ^1L_a state in all investigated solvents.

	**solvent**	**ε_r / F m^{-1}**	**NQMAm ^1L_a state / eV**	**PNQMA ^1L_a state / eV**
protic solvents	HFiP	16.7	4.11	3.92
	TCE	n.a.	4.04	3.88
	TFE	8.6	4.12	3.91
	H_2O	78.4	4.06	3.89
	MeOH	32.7	3.91	3.89
aprotic solvent	DCM	8.9	3.70	3.75

Table S2 Selected properties of the solvents used in this investigation.

solvent	HFiP	TCE	TFE	H_2O	MeOH	DCM
pK_a	9.3	12.2	12.4	14.0	15.5	-
ε_r / F m^{-1} a)	16.7[2]	n.a.	8.6[3]	78.4[4]	32.7[4]	8.9[3]
η / 10^{-3} Pa s b)	1.65	highly viscous*	1.75	0.91[4]	0.54	0.43
π^* c)	0.65	n.a.	0.73	1.09	0.60	0.82
β c)	0.00	n.a.	0.00	(0.18)	(0.62)	0.00
α c)	1.96	n.a.	1.51	1.17	0.93	(0.30)

a) dielectric constant b) solvent viscosity c) Kamlet-Taft parameters: π^* index of solvent dipolarity/polarizability, α hydrogen bond donor acidity, β hydrogen bond acceptor basicity,[5] , values in parentheses are according to ref. 27 relatively less certain, value not available (n.a.).

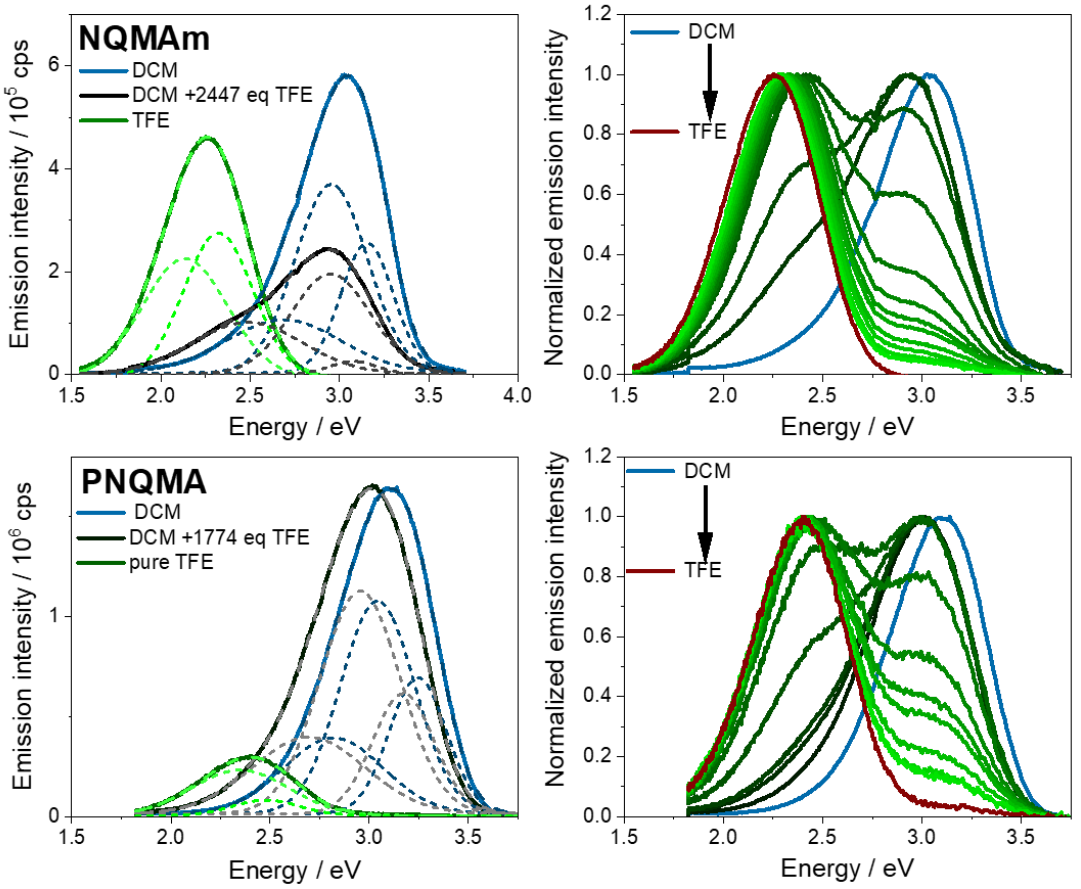

Figure S9 (Left panels) Emission spectra of the quinoline chromophore obtained in neat DCM, neat TFE and DCM with approx. 2000 eq. proton donor TFE (based on the quinoline concentration estimated *via* UV/vis measurements) and (right panel) normalized emission spectra obtained in the titration experiments: solvent = DCM, titrator = TFE, titrand = **NQMAm** and **PNQMA**.

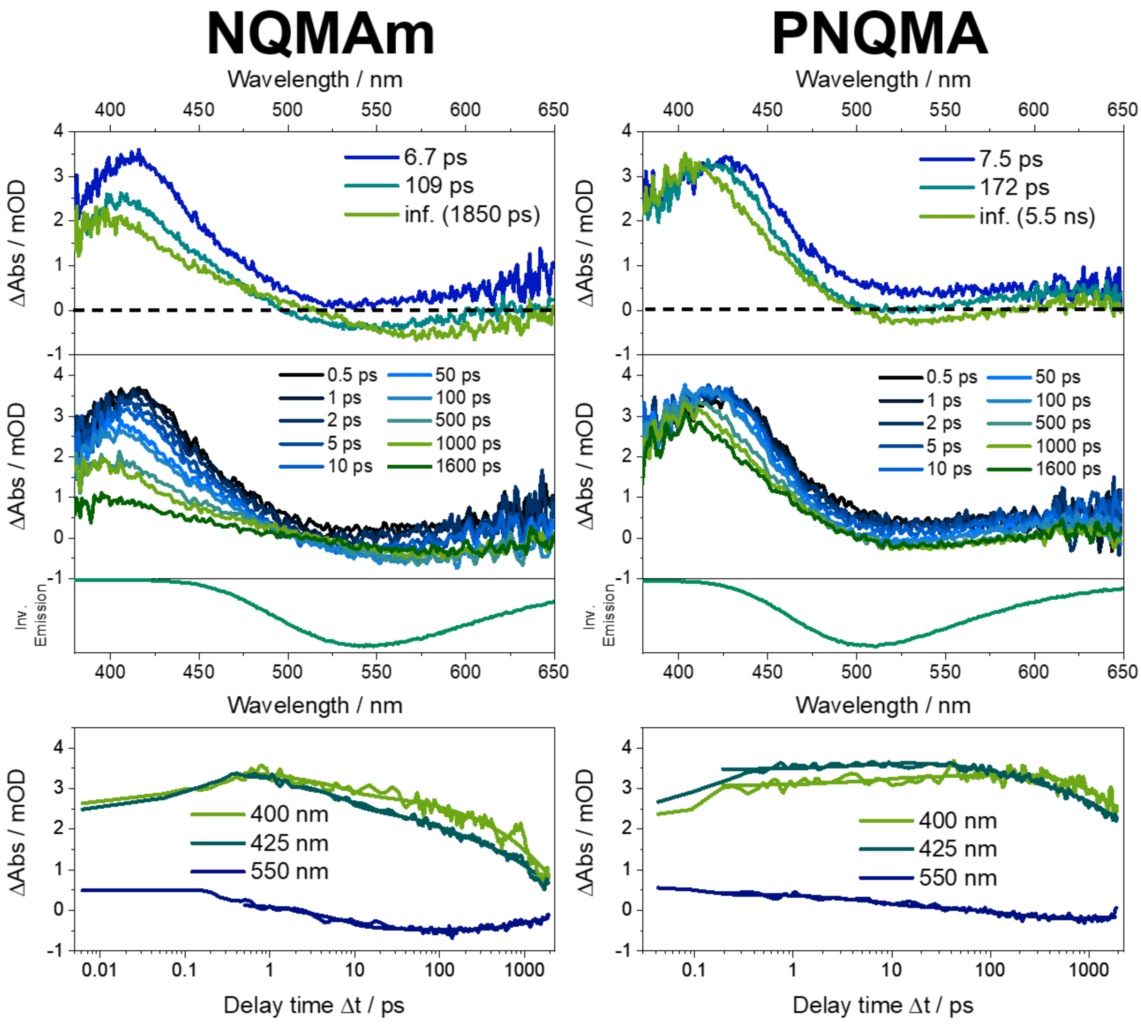

Figure S10 Compilation of *fs*-transient absorption data of **NQMAm** (left) and **PNQMA** (right) obtained in the solvent TCE (pK_a = 12.2, ES protonation of NQ). Respective upper panels of the graphs show the SADS and transient spectra obtained at selected delay times Δt after photoexcitation together with an inverted emission spectrum. The lower panels show kinetic traces at selected wavelengths.

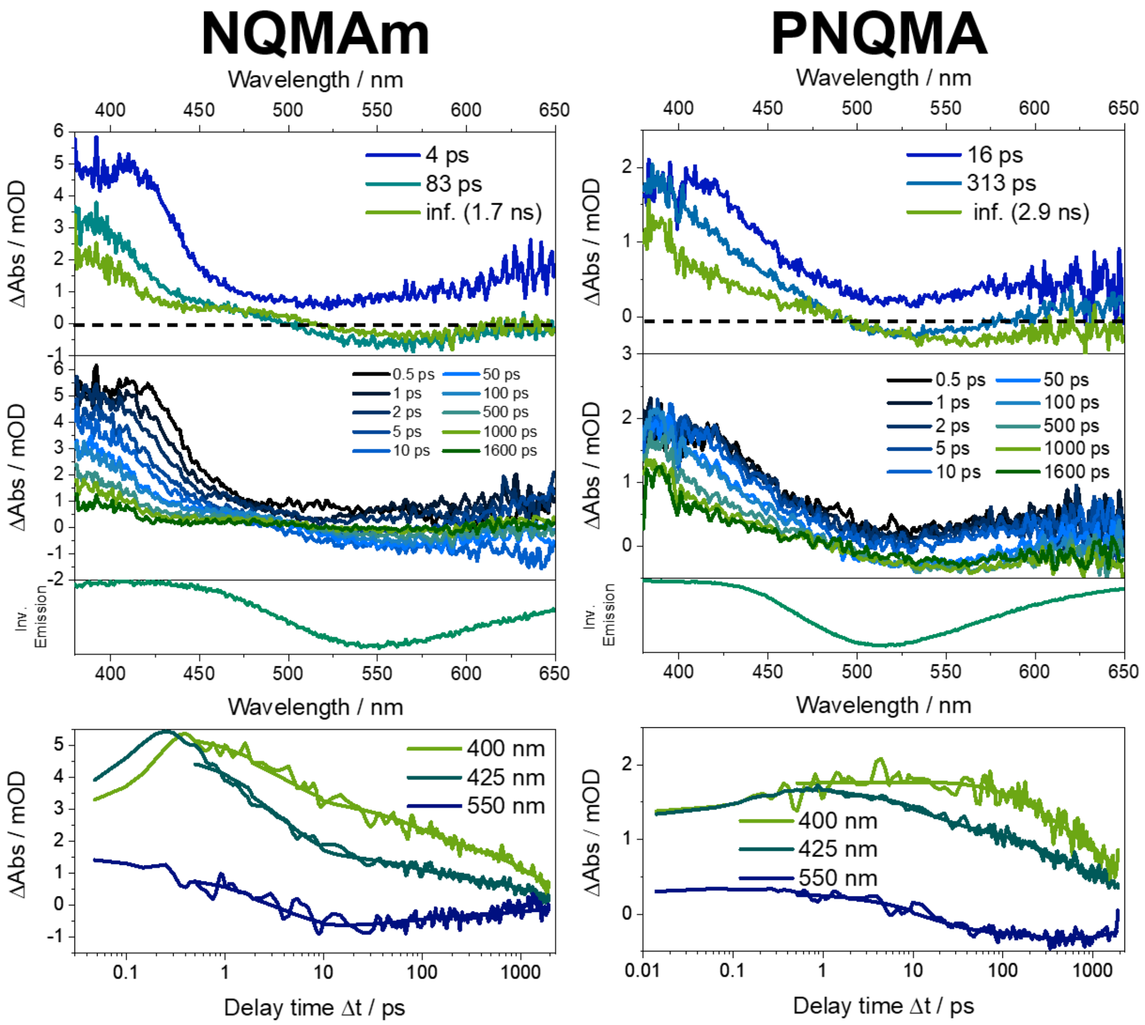

Figure S11 Compilation of *fs*-transient absorption data of **NQMAm** (left) and **PNQMA** (right) obtained in the solvent TFE (pK_a = 12.4, ES protonation of NQ). Respective upper panels of the graphs show the SADS and transient spectra obtained at selected delay times Δt after photoexcitation together with an inverted emission spectrum. The lower panels show kinetic traces at selected wavelengths.

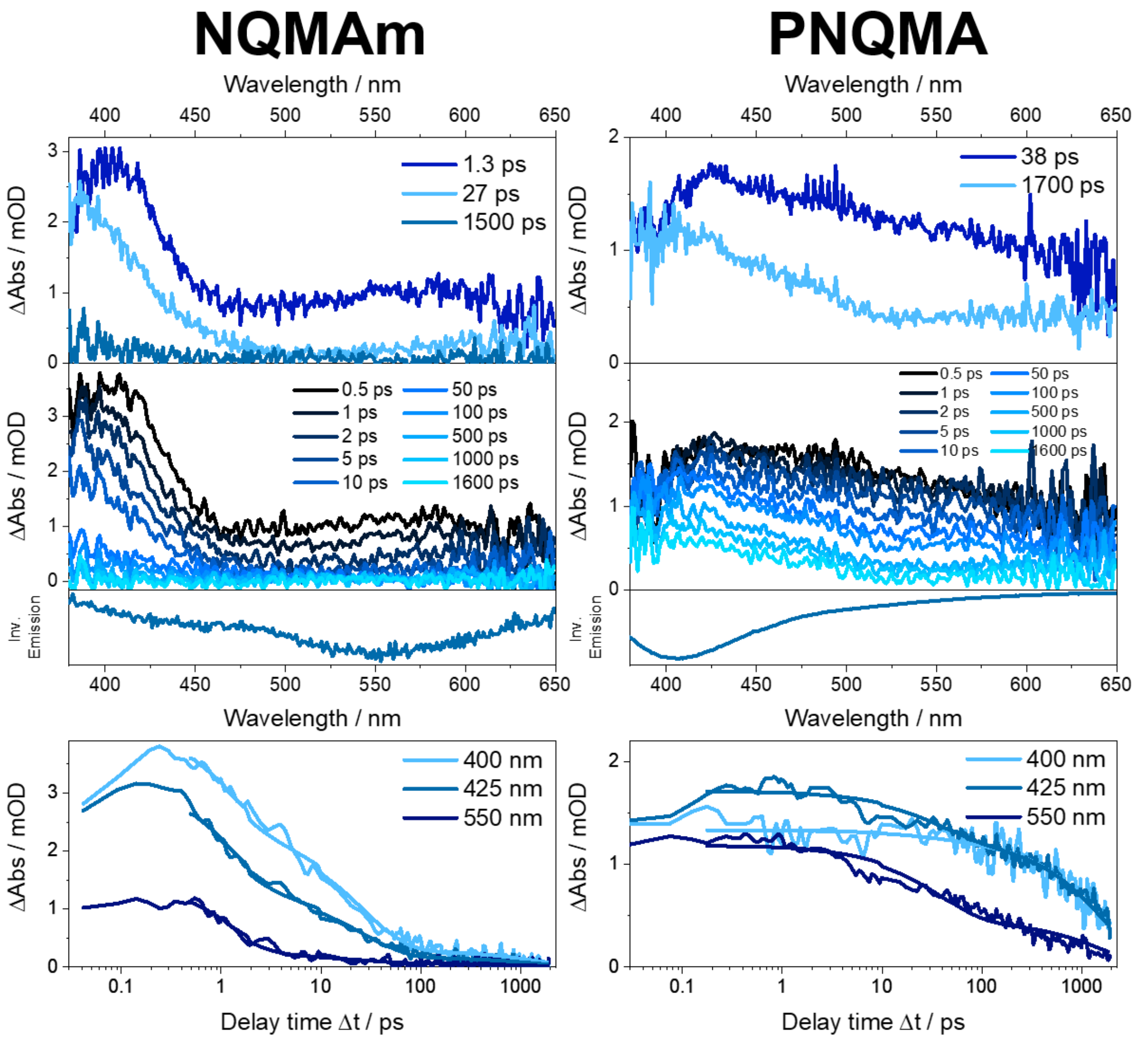

Figure S12 Compilation of *fs*-transient absorption data of **NQMAm** (left) and **PNQMA** (right) obtained in the solvent H_2O (pK_a = 14.0, no ES protonation of NQ). Respective upper panels of the graphs show the SADS and transient spectra obtained at selected delay times Δt after photoexcitation together with an inverted emission spectrum. The lower panels show kinetic traces at selected wavelengths. Contrary to the results obtained in the steady state emission of **NQMAm** in H_2O, the transient species associated with NQH could not be identified within the *fs*-TA studies. This may relate to the low thermodynamic driving force of the protonation reaction of 0.1 eV.

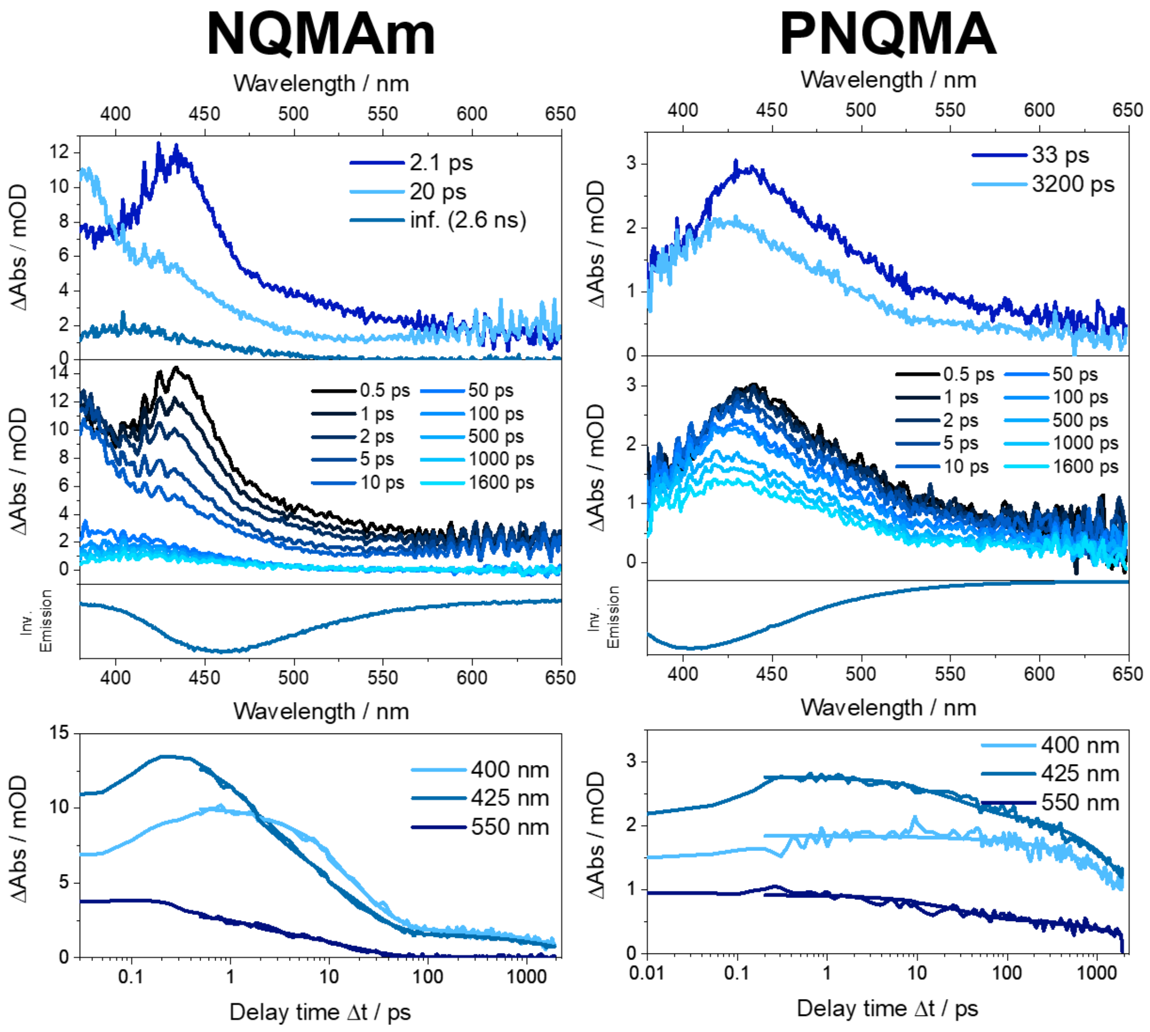

Figure S13 Compilation of *fs*-transient absorption data of **NQMAm** (left) and **PNQMA** (right) obtained in the solvent DCM (aprotic solvent, no ES protonation of NQ). Respective upper panels of the graphs show the SADS and transient spectra obtained at selected delay times Δt after photoexcitation together with an inverted emission spectrum. The lower panels show kinetic traces at selected wavelengths.

Table S3 Spectroscopic properties of **NQMAm** and **PNQMA** obtained in the solvents under investigation.

solvent		HFiP	TCE	TFE	H_2O	MeOH	DCM
**NQMAm**	ΔpK_a	4.8	1.9	1.7	0.1	(-1.4)	-
	λ_{abs}^{max} / nm	303 **NQ**	305 **NQ**	293 **NQ**	292 **NQ**	303 **NQ**	305 **NQ**
	ε_{abs}^{max} / 10^3 $M^{-1}cm^{-1}$	4.0	5.6	4.8	5.1	5.1	5.0
	λ_{em}^{max} / nm [E / eV]	536 [2.31] **NQH**	544 [2.28] **NQH**	549 [2.26] **NQH**	431 / 558 [2.88 / 2.22] **NQ+NQH**	420 [2.95] **NQ**	410 [3.07] **NQ**
	φ_{em}/ %	2.4	4.6	0.25	0.07	0.02	0.1
	τ_1/ ps	5	7	4	1	2	2
	τ_2/ ps	90	110	110	27	20	20
	τ_3/ ps	1500	1800	1700	-	-	Inf. (2.6 ns)
**PNQMA**	ΔpK_a	3.3	0.4	0.2	-1.4	-2.9	-
	λ_{abs}^{max} / nm	295 **NQ**	306 **NQ**	304 **NQ**	304 **NQ**	304 **NQ**	306 **NQ**
	λ_{em}^{max} / nm [E / eV]	507 [2.45] **NQH**	507 [2.45] **NQH**	507 [2.45] **NQH**	405 [3.06] **NQ**	407 [3.04] **NQ**	403 [3.08] **NQ**
	φ_{em}/ %	5.6	11.7	4.1	6.3	7.6	20
	τ_{em}/ ns	3.2	n.d.	2.1	1.5	2.4	5.0
	τ_1/ ps	12	8	20	40	32	33
	τ_2/ ps	140	180	200	1600 ps	inf. (2.3 ns)	inf. (3.1 ns)
	τ_3/ ps	inf. (3.3 ns)	Inf. (5.5 ns)	Inf. (3.0 ns)	-	-	-

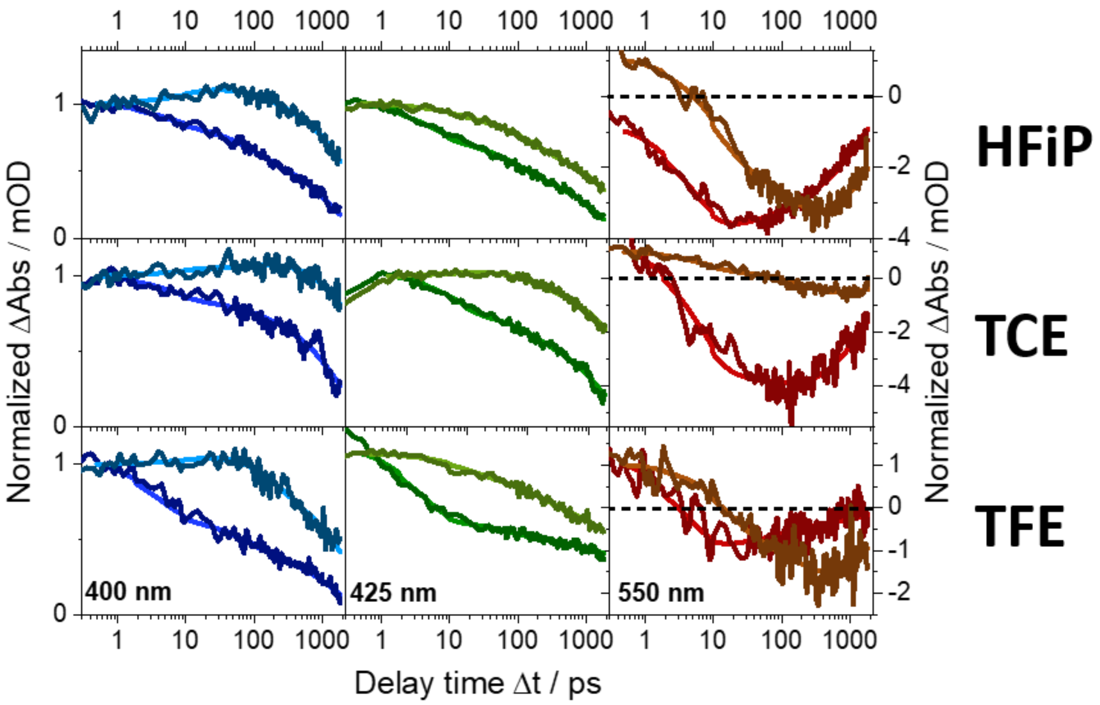

Figure S14 Normalized *fs*-TA kinetics at 400, 425 and 500 nm of **NQMAm** (light color) and **PNQMA** (dark color) in the solvents HFiP, TCE and TFE (ES protonation of NQ).

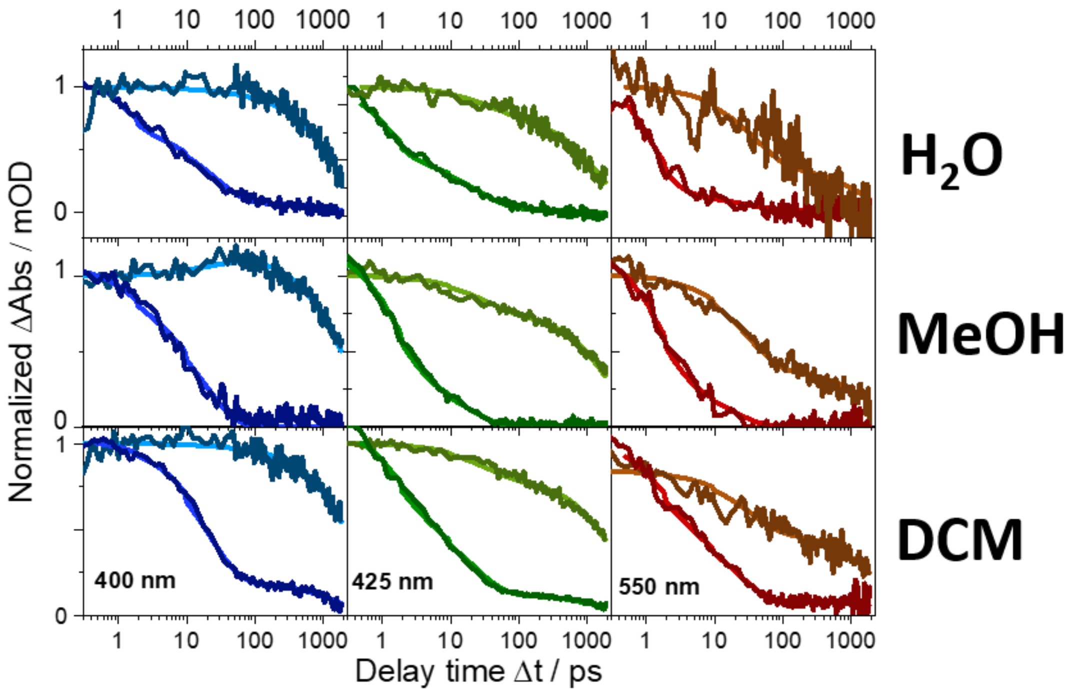

Figure S15 Normalized *fs*-TA kinetics at 400, 425 and 500 nm of **NQMAm** (light color) and **PNQMA** (dark color) in the solvents H_2O, MeOH and DCM (no ES protonation of NQ).

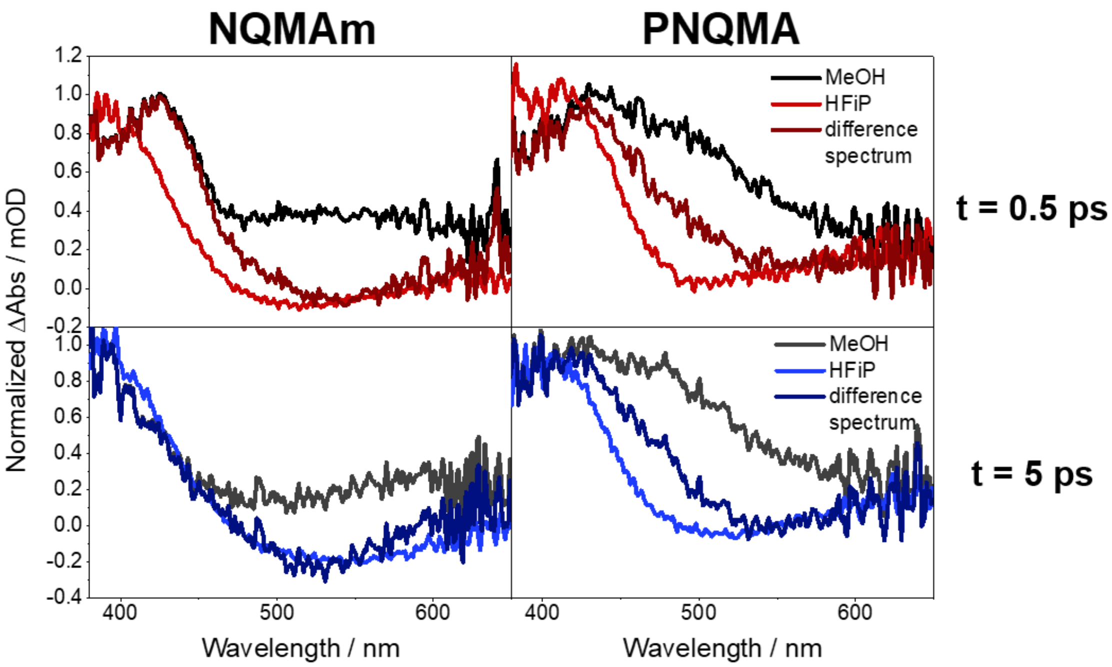

Figure S16 Normalized *fs*-TA spectra of **NQMAm** and **PNQMA** in the solvents MeOH (black) and HFiP (light colored spectra) obtained at delay times of t = 0.5 ps and 5 ps. Dark colored spectra show generated difference spectra of the respective MeOH spectrum and steady state emission spectra of protonated form NQH.

[1] T. Förster, *Zeitschrift für Elektrochemie und angewandte physikalische Chemie* **1950**, *54*, 42–46.
[2] M. Carraro, M. Gardan, A. Sartorel, C. Maccato, M. Bonchio, *Dalton Trans.* **2016**, *45*, 14544–14548.
[3] J. R. Hunt, C. Tseng, J. M. Dawlaty, *Faraday Discuss.* **2019**, *216*, 252–268.
[4] C. Reichardt, *Solvents and solvent effects in organic chemistry;* Wiley-VCH, Weinheim, **2005**.
[5] Mortimer J. Kamlet, Jose Luis M. Abboud, Michael H. Abraham, R. W. Taft, *J. Org. Chem.* **1983**, *48*, 2877–2887.
